# Integrin α2β1 inhibits MST1 kinase phosphorylation and activates Yes-associated protein oncogenic signaling in hepatocellular carcinoma

**DOI:** 10.18632/oncotarget.12760

**Published:** 2016-10-19

**Authors:** Kwong-Fai Wong, Angela M. Liu, Wanjin Hong, Zhi Xu, John M. Luk

**Affiliations:** ^1^ Department of Pharmacology, National University Health System, Singapore; ^2^ Department of Surgery, National University Health System, Singapore; ^3^ Institute of Molecular and Cell Biology, Biopolis, Singapore; ^4^ Department of Oncology, Nanjing First Hospital, Nanjing Medical University, Nanjing, China; ^5^ Department of Pathology, University of Hong Kong, Queen Mary Hospital, Pokfulam, Hong Kong; ^6^ Department of Translational and Clinical Medicine, Arbele Limited, Hong Kong Science Park, Shatin, Hong Kong

**Keywords:** extracellular stimuli, hippo pathway, liver cancer, MST1, Yes-associated protein

## Abstract

The Hippo pathway regulates the down-stream target Yes-associated protein (YAP) to maintain organ homeostasis, which is commonly inactivated in many types of cancers. However, how cell adhesion dysregulates the Hippo pathway activating YAP oncogene in hepatocellular carcinoma (HCC) remains unclear. Our findings demonstrate that α2β1 integrin (but not other β1 integrins) expressed in HCC cells, after binding to collagen extracellular matrix, could inhibit MST1 kinase phosphorylation and activate YAP pro-oncogenic activities. Knockdown of integrin α2 gene (*ITGA2*) suppressed YAP targeted gene expression *in vitro*. α2β1 and collagen binding resulted in suppressing Hippo signaling of mammalian sterile 20-like kinase 1 (MST1) and Large tumor suppressor homolog 1 (LATS1) with concomitant activation of YAP-mediated connective tissue growth factor (*CTGF)* gene expression. In vitro kinase assay showed that MST1 is an immediate downstream target of integrin α2 with S1180 residue as the critical phosphorylation site. Clinical correlational analysis using a gene expression dataset of 228 HCC tumors revealed that *ITGA2* expression was significantly associated with tumor progression, and co-expression with YAP targeted genes (*AXL receptor tyrosine kinase, CTGF, cyclin D1*, *glypican 3*, *insulin like growth factor 1 receptor, and SRY-box 4)* correlated with survivals of HCC patients. In conclusion, α2β1 integrin activation through cellular adhesion impacts the Hippo pathway in solid tumors and modulates MST1-YAP signaling cascade. Targeting integrin α2 holds promises for treating YAP-positive HCC.

## INTRODUCTION

Hepatocellular carcinoma (HCC) is a common cancer worldwide with over 700,000 new incidences per year [1, 2], and the major risk factor is associated with chronic hepatitis B infection [3]. Surgery remains the primary curative treatment for HCC for whom approximately 20% patients diagnosed in early stages, whereas the postoperative long-term survivals are still poor because of a high recurrence rate [4].

To dissect the genetic casualty of HCC initiation and progression, and to identify potential therapeutic target for HCC treatment, we employed a mosaic HCC mouse model and identified Yes-associated protein (YAP) as a bona-fide oncogene in liver cancer [5, 6]. YAP and its paralogue TAZ (transcriptional co-activator with PDZ-binding motif) are transcriptional co-activators regulated by the evolutionarily conserved Hippo pathway. The pathway components include two kinases named mammalian sterile 20-like kinase 1/2 (MST1/2), and Large tumor suppressor homolog 1/2 (LATS1/2).

Unlike that binding to extracellular matrix, cell-cell interaction activates MST1/2 thereby phosphorylating LATS1/2 using SAV1 as a cofactor. Activated LATS1/2 (in complex with Mob1) subsequently phosphorylates YAP at S127 and TAZ at S89, resulting in their cytoplasmic sequestration by 14-3-3 proteins [7-9] and proteosomal degradation of YAP/TAZ [10, 11]. During cell proliferation or organ regeneration (for instance, after partial hepatectomy) the Hippo pathway is inactivated, unleashing YAP and/or TAZ proteins for translocation into the nucleus, where YAP/TAZ protein interacts with transcriptional factors such as TEADs to “switch on” a wide range of genes involved in cell proliferation, de-differentiation, and survival [12]. In cancer cells the Hippo pathway is also inactivated, allowing nuclear YAP/TAZ to modulate genes for metastasis [13-16]. Our previous study on HCC clinical samples revealed aberrant nuclear YAP expression associated with the poor survival outcomes [17]. Recent study showed a cyclic-YAP peptide that disrupts YAP/TEAD interaction could inhibit HCC cell growth and survival [18].

YAP/TAZ are reported to regulate cell growth by sensing the cells toward the mechanical signals from extracellular matrix [19-22]. They are activated when cells are grown on a stiff matrix while inactivated when cells are grown on a soft matrix [23, 24]. Integrins are adhesion receptors in cell–matrix adhesion and α2β1 integrin was shown to induce MMP-7 expression through activating YAP independent of Hippo pathway [25]. α2β1 integrin of the β1-integrin family is an extracellular matrix receptor for collagen and laminin [26]. In addition to its physiologic function, α2β1 integrin contributes to the tumor-stromal interaction that is important to cancer cell survival and metastasis [27-29]. Herein, the present study reports α2β1 integrin as a potential upstream negative regulator of the Hippo pathway in HCC, which upon activation through binding to collagen, directly inhibits MST1 kinase and activates the YAP-driven transcriptional activities.

## RESULTS

### Integrin α2 is important for the expression of YAP targeted genes

**Figure 1:**
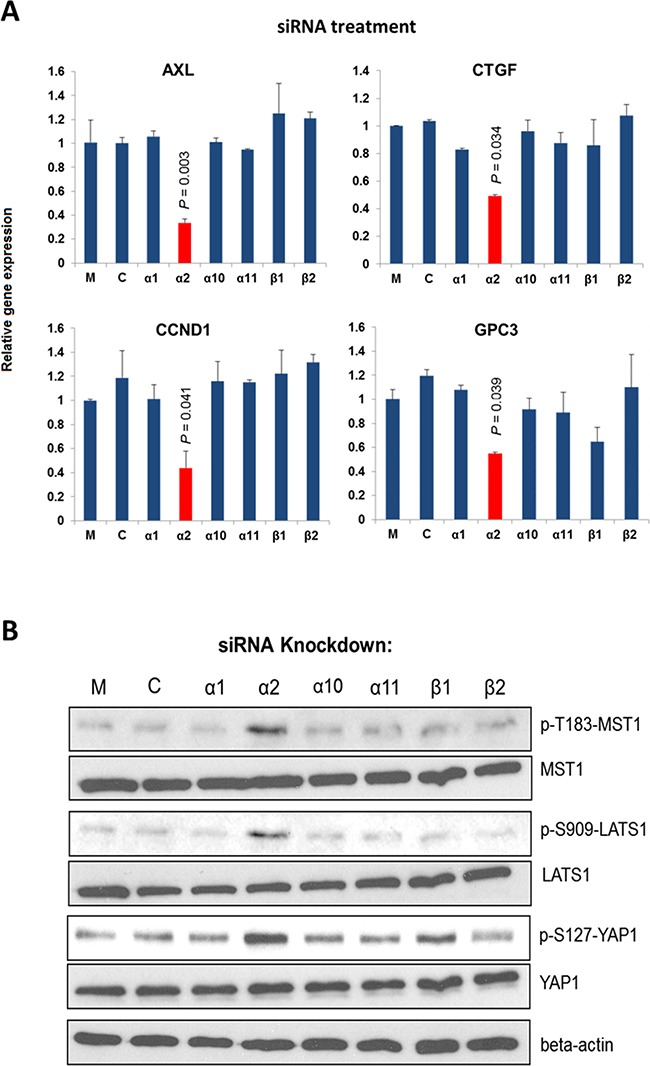
Integrin α2 is important for the expression of YAP targeted genes **A.** Knockdown of *ITGA2* in PLC cells suppressed significantly the expression of four YAP targeted genes (*AXL, CCND1, CTGF*, and *GPC3*) comparing with the mock-treated groups. **B.** Knockdown of *ITGA2* caused substantial increases in the phosphorylation at T183, S909, and S127 of MST1, LATS1, and YAP, respectively comparing with the groups treated with transfection reagent alone (*M*) and scrambled siRNA (*C*). All data shown are the representative of three independent experiments.

β1-integrin family functions as collagen receptors and consists of 4 members: α1β1, α2β1, α10β1 and α11β1. To determine which member is involved in regulating the Hippo pathway in HCC, we knocked down each individual subunit gene, and studied the changes in gene expression mediated by YAP activation (using the previously reported gene set including *AXL*, *CTGF*, *CCND1*, and *GPC3*) in HCC cells [30, 31]. Prior to the study the siRNAs were confirmed able to suppress respective integrin subunit expression by > 50% with no considerable off-target effects (Supplementary Figure S1). Results of experiment showed that knockdown of integrin α2 (*ITGA2*), but not other α subunits or β1 subunit, yielded significant suppression of the YAP targeted gene expression of PLC/PRF/5 (PLC) cells (Figure 1A). Similar knockdown effects could also be seen in Hep3B cells (Supplementary Figure S2).

We next investigated whether integrin α2 would sustain the expression of YAP targeted genes by modulating the Hippo core kinases MST1 and LATS1 [32]. As shown in Figure. 1B, knockdown of *ITGA2* led to substantial increases in p-T183-MST1, p-S909-LATS1, and p-S127-YAP levels, indicating an activated Hippo pathway with an inhibited YAP. By contrast, knockdown of integrin β1(*ITGB1*) or other pairing alpha subunits did not result in marked increase of the p-S127 YAP level. These findings suggest that integrin α2 plays a crucial and selective role in activating YAP by modulating the Hippo core kinases.

### Extracellular matrix (ECM) adhesion by α2β1 integrin inhibits the Hippo pathway

**Figure 2:**
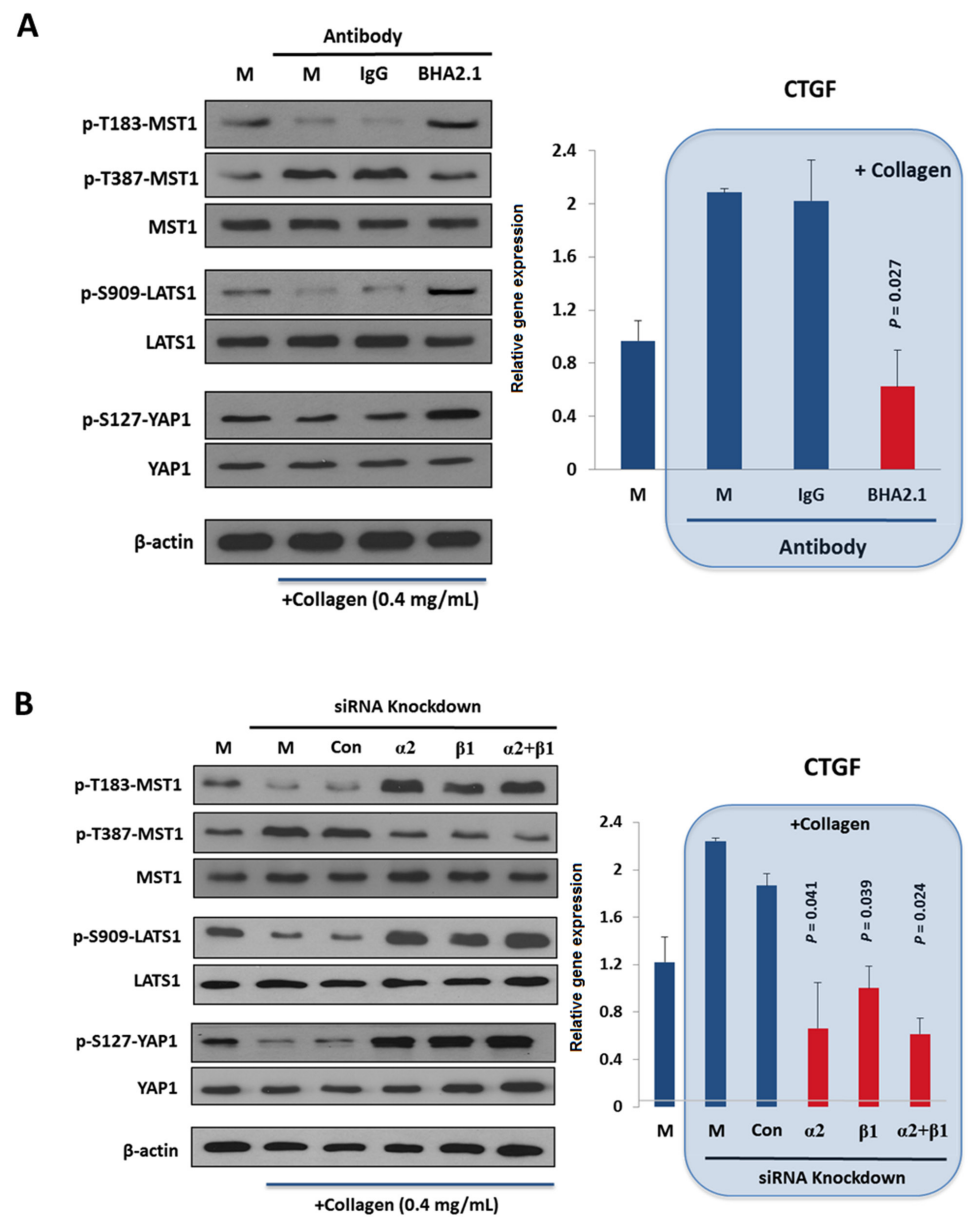
Extracellular matrix adhesion by α2β1 integrin adhesion inhibits the Hippo pathway **A.** Adhesion of Huh7 cells to collagen IV reduced the stimulatory p-T183-MST1 and p-S909-LATS1 but increased the inhibitory p-T387-MST1, leading to a decrease in p-S127-YAP and in turn YAP-mediated *CTGF* expressions (right panel). Treatment of the cells with an integrin function-blocking antibody (*BHA2.1*), but not PBS (*M*) and control IgG (*IgG*) antagonized the effect of collagen binding on Hippo pathway. **B.** Knockdown of *ITGA2* (*α2*) and *ITGB1* (*β1*) by siRNAs reversed the effect of collagen binding on Hippo pathway components, causing a significant reduction on CTGF expression comparing to the mock-treated group. All data shown are the representative of three independent experiments.

ECM plays important roles in regulating cell physiology such as cell division and migration [33]. Recently YAP was suggested as a transcriptional mediator (or mechano-sensor) for cellular responses toward ECM stiffness via a Hippo pathway-independent mechanism [23]. It is therefore of interest to examine whether binding of α2β1 integrin to ECM collagen would modulate the Hippo pathway in HCC cells. Unlike other HCC cell lines that were tested and found constitutively active, α2β1 integrin of Huh7 cells is present in the resting state that is responsive to collagen-dependent activation under normal physiologic conditions [34, 35]. As shown in Figure 2A (comparing lane 2 with lane 1), adhesion of Huh7 cells on collagen IV caused substantial decreases in the stimulatory phosphorylation at p-T183-MST1 and p-S909-LATS1, but increased the inhibitory phosphorylation at p-T387-MST1. Concomitant with these events, decrease of p-S127-YAP and up-regulation of the YAP targeted gene *CTGF* (right panel) were observed, implicating functional activation of YAP oncogene. Treatment of Huh7 cells with a functional blocker BHA2.1 antibody, which was shown to abolish the adhesive property of α2β1 integrin [36], could significantly suppress the collagen-induced *CTGF* expression that was accompanied by increases in p-T183-MST1 and p-S909-LATS1 (reactivation of MST and LATS) (Figure 2A, lane 4). BHA2.1 was generated through immunization of BALB/c mice with human fibrosarcoma HT1080 cells, and was shown able to bind α2β1 integrin transfectant cells in flow cytometry at 0.2 μg/mL [37].

In parallel, we demonstrated that knockdown of *ITGA2* and *ITGB1* could reverse the inhibitory effect of collagen IV on the Hippo pathway (Figure 2B). By contrast, the Hippo pathway of MIHA, a non-cancerous hepatocyte cell line, was not responsive to the collagen IV treatment (Supplementary Figure S3). Taken together, adhesion of α2β1 integrin to collagen IV was shown to inhibit the function of MST1 and LATS1, and thus activates the YAP transcriptional activity in HCC.

### MST1 is an immediate downstream target of integrin α2

**Figure 3:**
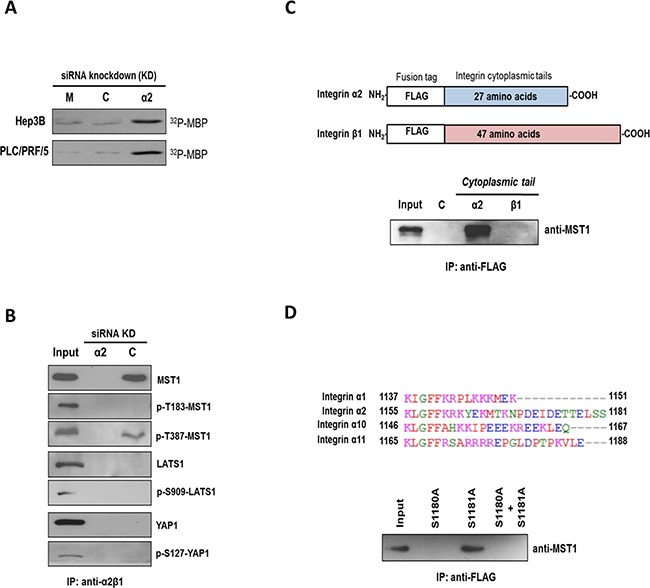
MST1 is an immediate downstream target of integrin α2 **A.** Endogenous MST1 was immunoprecipitated from Hep3B and PLC cells treated with control siRNA (C) or siRNA targeting *ITGA2* (*α2*) and subjected to in vitro kinase assay using myelin basic protein (MBP) as a substrate. Incorporation of radioactive ^32^P into MBP was higher in the siRNA-ITGA2 treated group comparing to mock and control siRNA-treated group. **B.** In scrambled control siRNA-treated Hep3B cells (*C*) α2β1 integrin was co-precipitated with MST1 and p-T387-MST1 but not p-T183-MST1. No co-precipitation was observed with YAP and LATS1 proteins. Cell lysate obtained from siRNA-ITGA2-treated Hep3B cells (*α2*) was also included as a negative control. **C.** The cytoplasmic tails of integrin α2 and β1 were cloned, and individually over-expressed in 293T cells as FLAG-tagged proteins (top). Endogenous MST1 could be pulled down with the cytoplasmic tail of integrin α2 but not β1 by the anti-FLAG affinity resin (bottom). **D.** Multiple sequence alignment showed integrin α2 differs from α1, α10, and α11 at its carboxyl end with two additional serine residues S1180 and S1181 (top). Integrin α2 cytoplasmic tail mutants with single mutation (i.e. S1180A or S1181A) and double mutations (i.e. S1180A + S1101A) were over-expressed in 293T cells as FLAG-tagged proteins. Endogenous MST1 was not pulled down with the FLAG-tagged protein with S1180A mutation by the anti-FLAG affinity resin (bottom). All data shown are the representative set of two independent experiments.

*In vitro* kinase assay showed that knockdown of *ITGA2* resulted in marked increases in the kinase activity of MST1 in Hep3B and PLC cells (Figure 3A). Furthermore, α2β1 integrin was selectively co-precipitated with MST1 and p-T387-MST1 (inactive form) but not LATS1 and YAP (Figure 3B). To map the MST1-binding site(s) on α2β1 integrin, the cytoplasmic tails of each integrin subunit were expressed in 293T cells as chimeric FLAG-tagged proteins for co-immunoprecipitation (Figure 3C, top). MST1 was precipitated with the cytoplasmic tail of integrin α2 but not the β1 subunit by anti-FLAG tag affinity resin (Figure 3C, bottom). Homologous protein sequence alignment revealed that the cytoplasmic tail of integrin α2 differs from other integrin counterparts α1, α10, and α11 at the S1180 and S1181 residues (Figure 3D, top). To examine whether the serine residues would be essential to the interaction between integrin α2 and MST1, we showed that substitution of S1180 with alanine prevented the co-precipitation of FLAG-tagged protein with MST1 (Figure 3D, bottom). These findings indicated MST1 is an immediate downstream target of α2β1 integrin, with MST1 binding to the cytoplasmic tail of integrin α2.

### FAK mediates α2β1 integrin inhibitory action on the Hippo pathway

**Figure 4:**
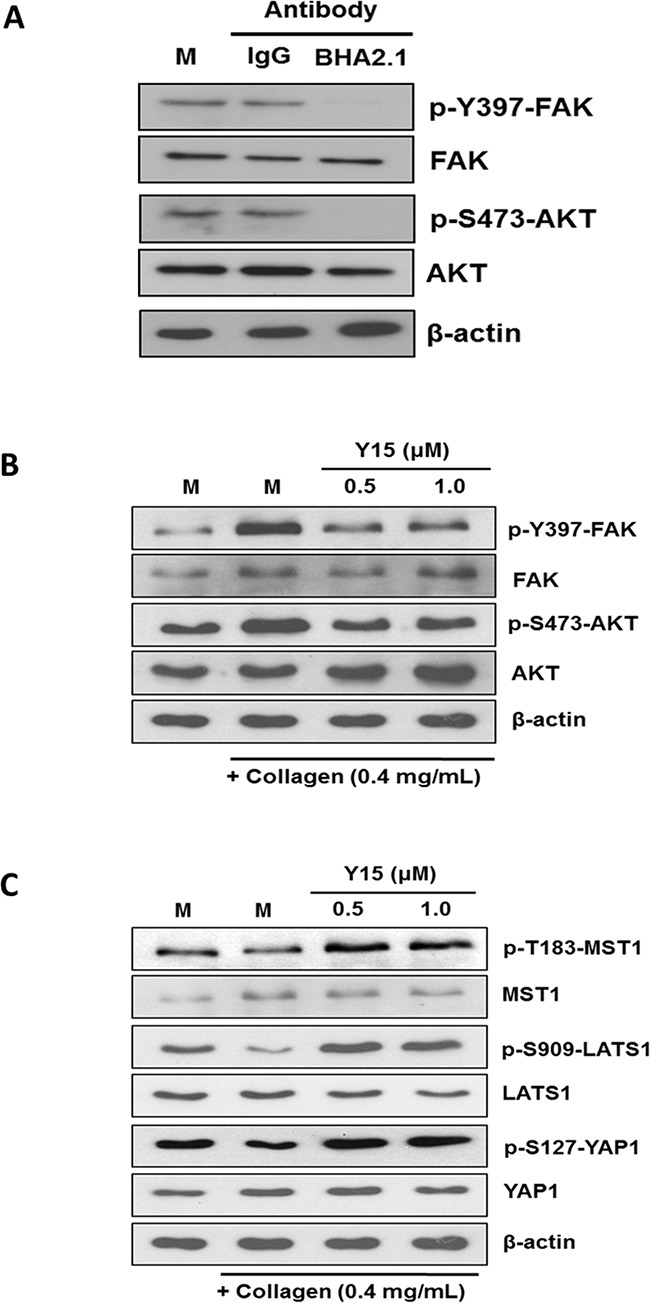
FAK mediates the action of integrin α2 on the Hippo pathway **A.** Hep3B cells were treated with sterile PBS alone (*M*), isotype-control IgG (*IgG*), and integrin function blocking antibody BHA2.1 (*BHA2.1*). Blocking the function of α2β1 integrin was demonstrated to decrease the stimulatory phosphorylation at Tyr397 and S417 of FAK and AKT, respectively. **B.** The effect of Y15 on FAK and AKT was studied in Huh7 cells cultured on immobilized collagen. Western blotting showed that collagen induced stimulatory phosphorylation of FAK and AKT at Y397 and S473, respectively. Treatment of Huh7 cells with 0.5 and 1.0 μM Y15 decreased FAK and AKT phosphorylation to the levels comparable to those of the un-inactivated FAK and AKT. **C.** FAK inhibition was shown to abrogate the collagen regulation of the Hippo pathway. Comparing to the mock control, Huh7 cultured on immobilized collagen showed lower levels of p-T183-MST1, p-S909-LATS1, and p-S127-YAP1 of Huh7. Oppositely, treatment of Huh7 cells with 0.5 and 1.0 μM Y15 could up-regulate the phosphorylation of MST1, LATS1 and YAP1. The effects were comparable to BHA2.1 blocker and integrin α2 siRNA treatment. All data shown are representative western blotting of two independent experiments.

Focal adhesion kinase (FAK) plays important roles on integrin-ECM mediated adhesion signaling events [38]. We demonstrated herein that treatment of Hep3B cells with BHA2.1 blocker mAb abolished the stimulatory phosphorylation at Y397 of FAK and S473 of AKT (Figure 4A). To investigate whether FAK would mediate the integrin α2-MST1 signaling axis in response to collagen adhesion, we showed that treatment of Huh7 with FAK inhibitor Y15 could suppress the collagen-induced FAK and AKT phosphorylation (Figure 4B). Along this line, we further showed that Y15 treatment could up-regulate the levels of p-T183-MST1, p-S909-LATS, and p-S127-YAP (Figure 4C), suggesting FAK inhibition blocked integrin α2-mediated inhibition of the Hippo pathway.

### An integrin α2-activated YAP oncogenic signature predicts prognostic outcomes in HCC patients

**Figure 5:**
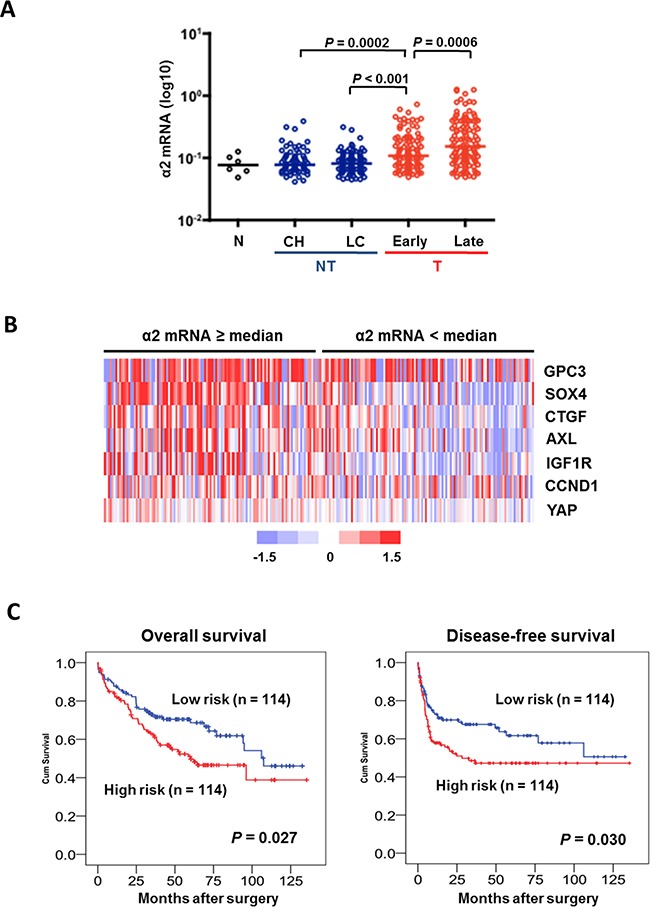
An integrin α2-activated YAP oncogenic signature predicts prognostic outcomes in HCC patients **A.** Analysis of transcriptome data of 228 HCC patients showed *ITGA2* expression was elevated in HCC tumors comparing to non-neoplastic lesions like chronic hepatitis (CH) and liver cirrhosis (LC). *ITGA2* expression in late-stage tumor was also higher than in the early staged. **B.** Expression of a set of YAP-targeted genes (*AXL, CCND1, CTGF, GPC3, IGF1R, and SOX4*) was found higher in patients over-expressing integrin α2 (i.e. mRNA ≥ medium), comparing to patients with integrin α2 expression < medium. Red, over-expression; blue, under-expression; white, no changes. **C.** Kaplan-Meier analysis illustrated the significant difference in overall (*P* = 0.027) and disease-free (*P* = 0.03) survivals between patients with high (≥ medium) and low (< medium) co-expression of *ITGA2* and YAP-targeted gene set.

Our analysis on cDNA microarray data derived from 228 patients with HBV-related HCC demonstrated a stepwise increase in *ITGA2* expression from non-neoplastic lesions (i.e. chronic hepatitis and cirrhosis) to early HCC and from early to late HCC (Figure 5A). Furthermore, as revealed by hierarchical clustering *ITGA2* co-expressed with YAP targeted genes (including *AXL, CCND1, CTGF, GPC3, IGF1R, SOX4*) in HCC samples (Figure 5B). The co-expression could also be seen in a separate cohort of 24 HCC patients by RT-qPCR (Supplementary Figure S4). To test the clinical significance of the co-expression, we generated a logistic regression model combining *ITGA2* and YAP targeted genes, and determined the risk of co-expression of each patient. Kaplan-Meier analysis suggested the correlation of high risk score (i.e. ≥ median) with shorter overall (*P*=0.027) and relapse-free (*P*=0.030) survivals after hepatic surgery (Figure 5C). The risk score was associated with different clinicopathological parameters as well (Table 1).

**Table 1:** Clinical pathological correlation analysis of YAP-activated gene set with prognostic outcomes in HCC patients (n=228)

Variables	Frequency (%)	Risk score		
		< Median	≥ Median	*P* values
Sex				
Male	179 (78.5)	97	82	***0.016***
Female	49 (21.5)	17	32	
Age, years				
< 55	105 (46.1)	38	67	***< 0.001***
≥ 55	123 (53.9)	76	47	
Alcohol *(n = 217)*				
No	133 (61.3)	66	67	0.907
Moderate/Heavy	84 (38.7)	41	43	
Alpha fetoprotein, ng/mL				
< 100	116 (50.9)	82	34	***< 0.001***
≥ 100	112 (49.1)	32	80	
HBsAg				
Negative	31 (13.6)	17	14	0.562
Positive	197 (86.4)	97	199	
Tumor size, cm				
< 5	77 (33.8)	37	40	0.674
≥ 5	151 (66.2)	77	74	
TNM stage				
Early (I, II)	103 (45.2)	60	43	**0.029**
Late (III, IV)	125 (54.8)	53	72	
Histological differentiation *(n = 191)*				
Well	37 (19.4)	26	11	***0.022***
Moderate/Poor	154 (80.6)	76	78	
Venous infiltration *(n = 227)*				
Absent	114 (50.2)	68	46	***0.007***
Present	113 (49.8)	45	68	
Tumor recurrence				
Absent	108 (47.4)	62	46	***0.034***
Present	120 (52.6)	52	68	

## DISCUSSION

**Figure 6:**
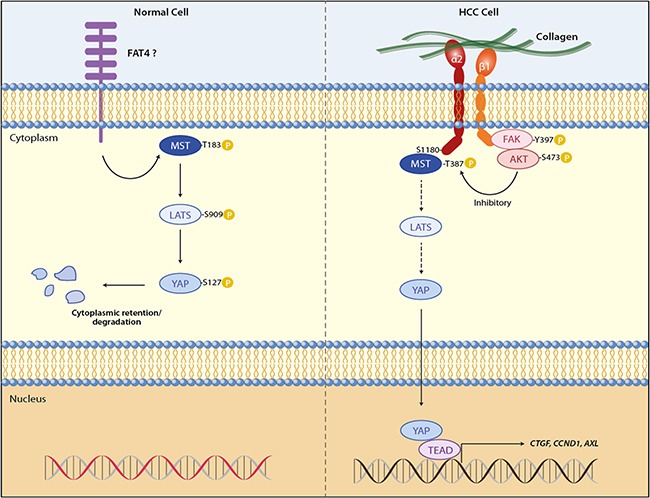
ECM adhesion of α2β1 integrin promotes YAP-mediated transcription through inhibition of MST1 of the Hippo pathway In normal cells MST1 and LATS1 activated by extracellular signals like cell-cell contact phosphorylate YAP at S127, retaining YAP in cytoplasm for sequestration and degradation. This suppresses YAP targeted gene expression, allowing the cell to maintain homeostasis. In liver cancer cells adhesion of α2β1 integrin to extracellular collagen inhibits the kinase activity of MST1 and subsequently LATS1, of which the molecular mechanism involves the interaction between MST1 and S1180 of integrin α2. YAP is released from the negative regulation of the Hippo core kinases, and is transported into the nucleus to activate gene transcriptions for cell proliferation and survival, helping cancer cells overcome the cell-cell contact inhibition.

Cell-extracellular matrix interaction shows substantial impacts on cell fate because it is one of the major mechanical cues to regulate organ development and homeostasis [39, 40], and on the other hand, its pathological perturbations contribute to malignant progression by inducing cytoskeleton remodeling [41]. YAP and TAZ, the transcriptional co-activators regulated by the Hippo pathway, have been identified as effectors in response to ECM elasticity and cell shape, of which the mechanism involves the control of both co-activators by F-actin and stress fiber [20, 21, 42]. Mechanosensitive phenotypes can also be regulated by integrins and their association with YAP/TAZ. For example, the integrin-linked kinase (ILK) induced YAP/TAZ nuclear localization through suppression of the Hippo pathway via phospho-inhibition of MYPT1-PP1 and inactivation of Merlin [43]. Stiff substrates enhanced colorectal cancer cell viability by upregulating MMP-7 expression through a positive feedback loop containing YAP, EGFR, integrin-α2β1 and MRLC, independent of Hippo pathway [25]. α2β1 integrin can directly interact with CDH17 through its RGD motif [44], which causes β1 integrin activation and signaling to induce focal adhesion kinase and Ras activation, leading to colorectal cancer cells proliferation and liver metastasis [28]. Despite these important findings, the cell-surface components upstream the Hippo pathway and that sense the pathway to extracellular stimuli, especially in the context of pathogenesis like carcinogenesis, have remained to be elucidated. To address this unresolved question, the present work demonstrates that the binding of α2β1 integrin to collagen IV, which is the ECM that is highly accumulated in tumor interstitium [45, 46], inhibits the Hippo core kinases MST1 and LATS1, liberating YAP for its transactivation of gene expression in the nucleus (Figure 6). Our transcriptome analysis on HCC patients shows a stepwise increase in *ITGA2* expression along the hepatocellular carcinogenic process, and patients co-expressing *ITGA2* and YAP-targeted genes including *AXL, CCND1, CTGF, GPC3, IGF1R, SOX4*, were found to show more dismal prognosis after hepatic surgery. Thus, it is believed that HCC cells adherent to ECM collagen in tumor microenvironment capitalize α2β1 integrin to modulate the Hippo tumor suppressor functions thereby overcoming the general physiology of contact-contact inhibition.

Loss of MST1 activity results in tumor nodules formation in mouse livers [47], however the inactivation of MST1 in human HCC remains to be fully elucidated, although few regulators of MST1 have been proposed [48-51]. Here we show for the first time that in HCC cells MST1 is a direct downstream target of α2β1 integrin. MST1 kinase activity is inhibited upon the binding of integrin receptor to ECM collagen. Our work suggests the inhibition of MST1 by α2β1 integrin depends on the physical interaction between the cytoplasmic tail of integrin α2 and MST1 kinase. Since integrin α2 selectively interacts with the inactivated p-T387-MST1, it is possible that the binding of integrin α2 to MST1 would prevent the dephosphorylation of p-T387 of MST1 by phosphatase, which is required for the full activation of MST1 [50]. The integrin β1-linked FAK/AKT may also be involved in the MST1 inhibition by α2β1 integrin because the effect of collagen on MST1 and LATS1 phosphorylation can be reversed by FAK inhibitor treatment.

Cytoplasmic tails of integrin α subunits are essential in determining the integrin-mediated downstream signaling [52-54]. Our present study identifies S1180 resident at the α2 cytoplasmic tail having a critical role for the binding of integrin α2 to MST1. As shown by sequence alignment the residue is exclusively present in integrin α2, but absent at the respective positions at integrin α1, α10, and α11 of other collagen receptor integrins. This finding may explain why only knockdown of integrin α2 can reduce YAP-mediated gene expression, and strengthens the unique functional role of α2β1 integrin in HCC. Therapeutic options for patients with advanced HCC are still limited. It is of great interest to investigate whether interrupting the interaction between integrin α2 and MST1 would show therapeutic potential for treatment of YAP-positive HCC.

## MATERIALS AND METHODS

### Antibodies, FAK inhibitor, cell culture, and transfection

MST1 (rabbit polyclonal, #3682), p-T183-MST1 (rabbit polyclonal, #3681), LATS1 (rabbit monoclonal, #3477), p-S909-LATS1 (rabbit polyclonal, #9157), p-S127-YAP (rabbit polyclonal, #4911), FAK (rabbit polyclonal, #3285), p-Y397-FAK (rabbit polyclonal, #3283), AKT (rabbit polyclonal, #9272), and p-S473-AKT (rabbit monoclonal, #3787) antibodies were purchased from Cell Signaling Technology (Danvers, MA). Endogenous YAP (rabbit polyclonal, #PAB9591) antibody was purchased from Abnova (Taipei, Taiwan). Anti-serum against p-T387-MST1 was from Prof. Ye of the Emory University School of Medicine (Atlanta, GA). FAK inhibitor Y15 was from Selleck Chemicals (Houston, TX). Hepatoma cell lines Huh7, Hep3B and PLC were obtained and cultured as described [55]. All siRNAs including negative controls (20 μM) (Life Technologies, Carlsbad, CA) were transfected using Lipofectamine RNAiMax (Life Technologies).

### Integrin subunit cDNA constructs and site-directed mutagenesis

The cDNA fragments of the cytoplasmic tails of integrin α2 and β1 were cloned into pci-puro-MCS-FLAG vector through *EcoRI* and *NotI* restriction sites, allowing the pull-down of the N-terminal FLAG-tagged proteins by anti-FLAG affinity gel. Overlapping PCR was done as described to substitute S1180 and S1181 of integrin α2 with alanine [56].

### Collagen binding experiment

Huh7 cells were seeded on culture plates coated with 0.4 mg/mL collagen IV (Sigma & Co., St. Louis, MO) and one day later, were treated with siRNAs: control, integrin α2 or β1 siRNAs; or antibodies: mouse IgG or α2β1 integrin function-blocking antibody BHA2.1 (mouse monoclonal, #MAB1998Z, Millipore, Temecula, CA). After 48 hours, cell lysates were obtained for western blotting as described [57].

### In vitro assays

Real-time PCR [57] and immunoprecipitation [58] were done as previously reported. To perform in vitro kinase assay, whole-cell lysate from Hep3B and PLC cells were precipitated by MST1 antibody (Cell Signaling). The washed precipitates were then used in the kinase assay. Briefly, reactions were performed in the presence of 10 μCi of [γ-32P] ATP in 30 μl assay buffer (20 mM HEPES, pH 7.4, 10 mM MgCl2, and 1 mM dithiothreitol) using myelin basic protein (MBP, Millipore, Temecula, CA) as the substrate. Reaction mixture was incubated at room temperature for 30 minutes, then stopped by adding SDS-PAGE sample buffer, and finally resolved by electrophoresis. After that, the incorporation of radioactive ATP into MBP was determined by autoradiography.

### Statistical analysis

All values were expressed as the mean ± S.D and statistical significance was evaluated using GraphPad PRISM for Macintosh (version 4.0; GraphPad, La Jolla, CA). The differences between all groups were evaluated by the Student's *t* test. Expression data of integrin α2 and YAP targeted genes were retrieved from our previous profiling study on HBV-related HCC, of which the raw data have been deposited in GEO with accession number GSE25097 [59, 60]. Survival analysis was performed using IBM SPSS Statistics version 19 (SPSS Inc., Chicago, IL). For Cox analysis, the regression coefficients of integrin α2 and YAP-targeted genes were first determined, and then be used for risk score calculation using the following formula: Risk score of each patients = (*ITGA2*)*0.275 + (*GPC3*)*0.42 + (SOX4)*0.587 + (*CTGF*)*0.389 – (*CCND1*)*0.05 – (*AXL*) *0.496 – (*IGF1R*)*0.086. Hierarchical clustering and cluster visualization was performed using Gene Cluster 3.0 and TreeView version 1.1.3, respectively.

## SUPPLEMENTARY MATERIALS FIGURES


