# Supplementary material for: Integrin α2β1 inhibits MST1 kinase phosphorylation and activates Yes-associated protein oncogenic signaling in hepatocellular carcinoma
Source: Oncotarget. 2016 Oct 19;7(47):77683–95. doi: 10.18632/oncotarget.12760 (PMC5363613; doi:10.18632/oncotarget.12760)
Supplement: Supplementary file 1 [file oncotarget-07-77683-s001.pdf]

Integrin α2β1 inhibits MST1 kinase phosphorylation and activates Yes-associated protein oncogenic signaling in hepatocellular carcinoma

SUPPLEMENTARY FIGURES

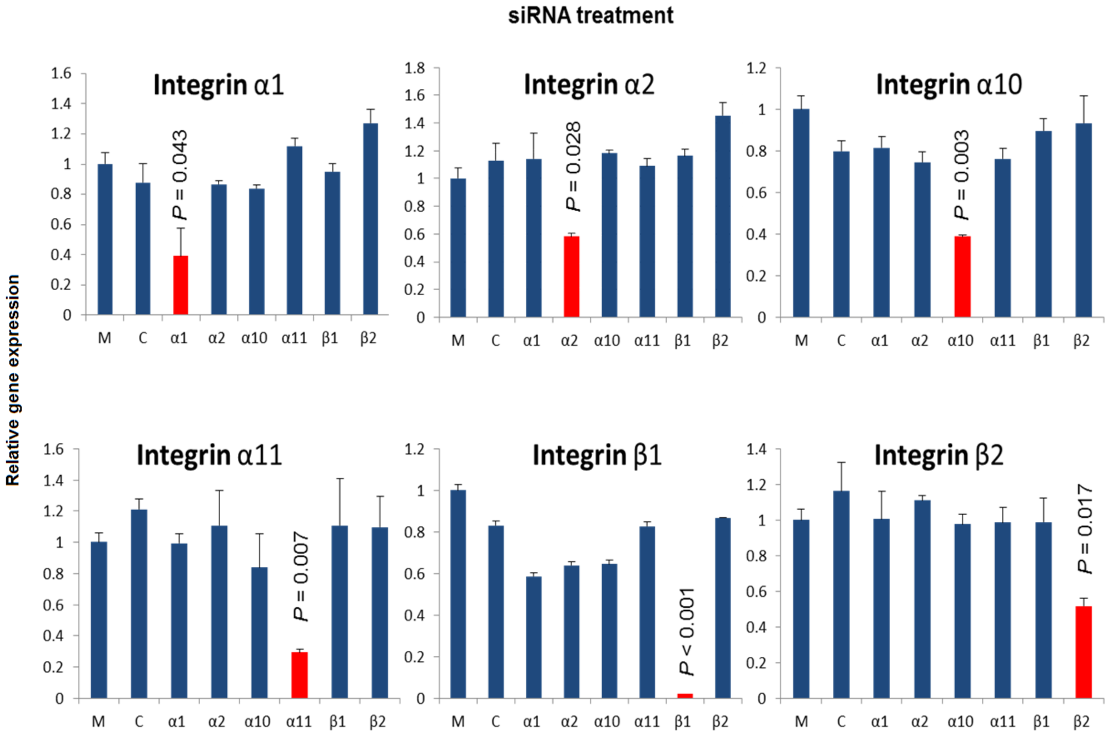

Supplementary Figure S1: Knockdown efficacies and potential off-target effects of integrin siRNAs used in this study were assessed using real-time PCR. Treatment of HCC cell lines with siRNAs suppressed respective integrin subunit expressions by > 50% with no considerable off-target effects. *P* values represent the significance of the comparisons between the mock and integrin siRNA-treated group.

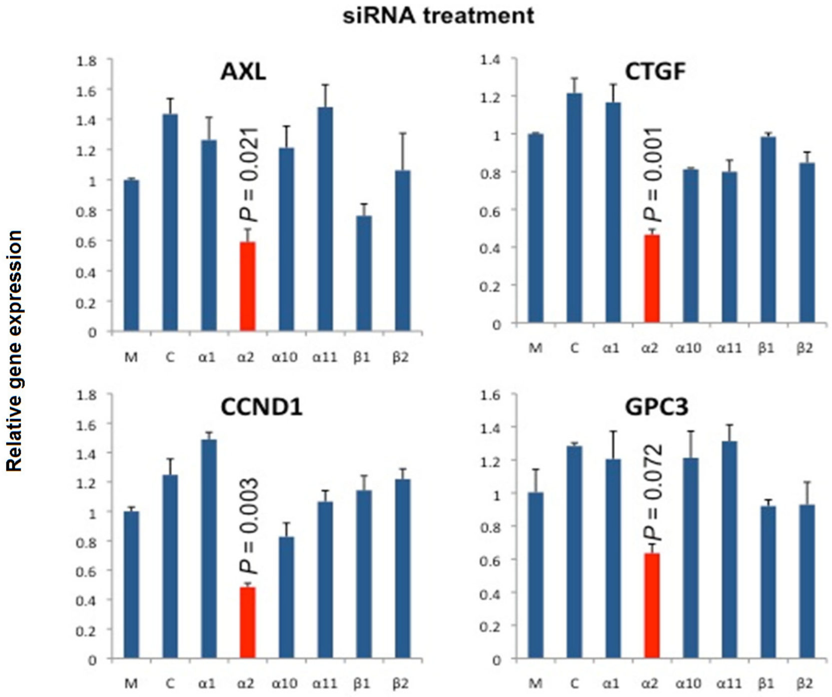

Supplementary Figure S2: Knockdown of *ITGA2* in Hep3B cells suppressed the expression of four YAP targeted genes (*AXL, CCND1, CTGF,* and *GPC3*) comparing with the mock-treated groups. All data shown are the representative of three independent experiments.

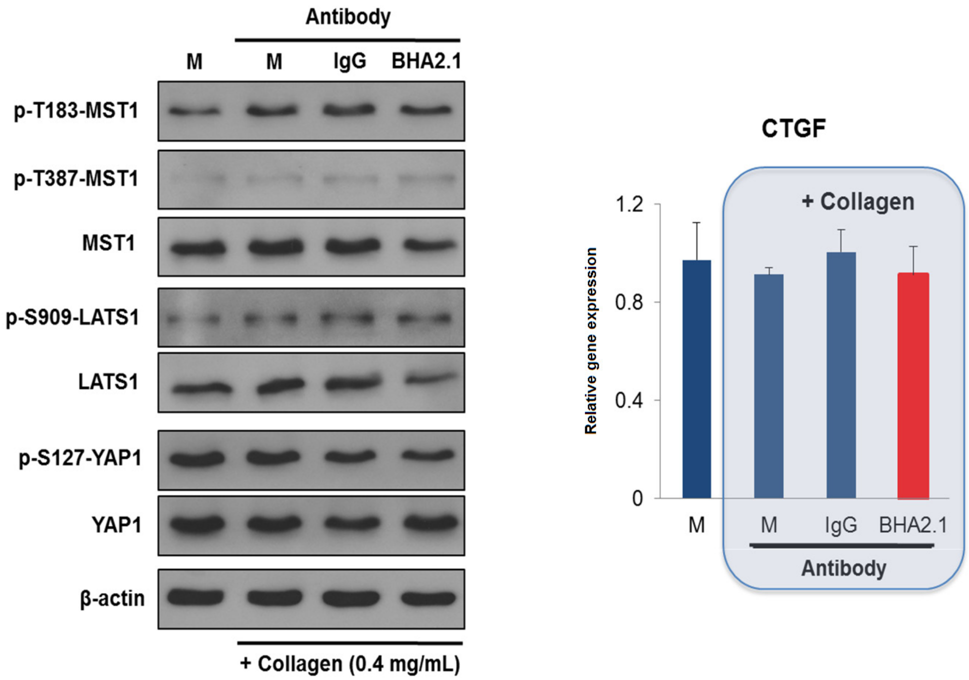

Supplementary Figure S3: Effect of collagen IV on the Hippo pathway was also studied in immortalized noncancerous hepatocytes MIHA cells. MIHA cells were cultured on 6-well plates coated with collagen IV, and were treated with sterile PBS alone (*M*), control IgG (*IgG*) or an integrin function-blocking antibody (*BHA2.1*). Unlike the situation observed in Huh7, neither the Hippo pathway phosphorylation nor CTGF expression was affected by the binding of MIHA cells to collagen.

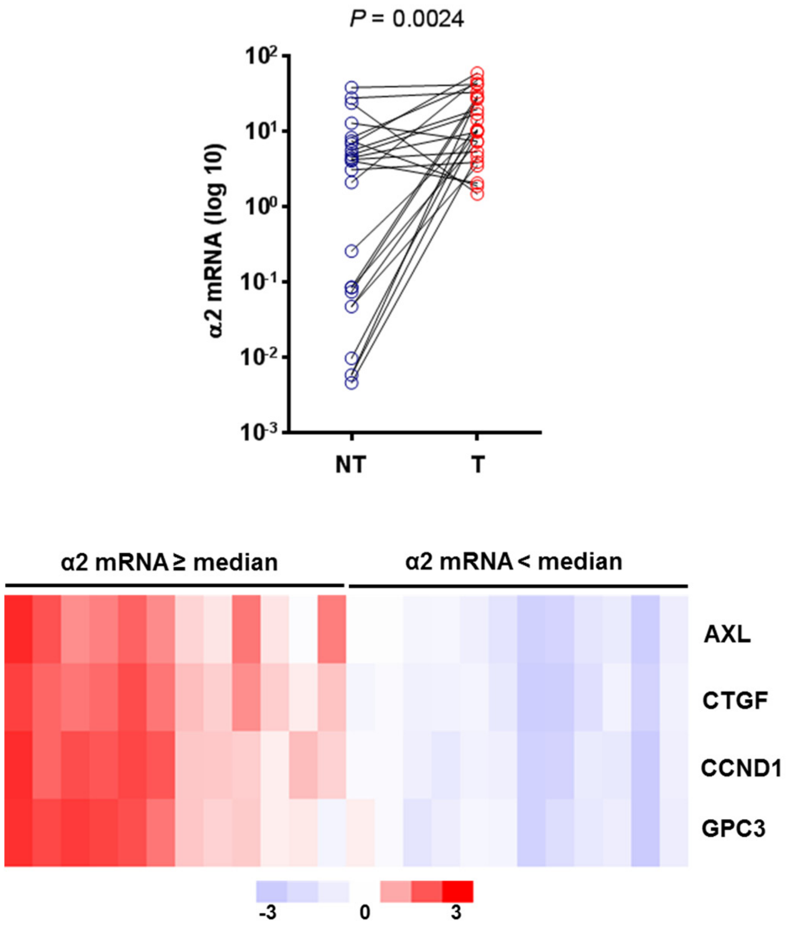

Supplementary Figure S4: The overexpression of integrin α2 in HCC was validated in an independent cohort of 24 patients using real-time PCR (top panel). Hierarchical analysis on the data showed expression levels of AXL, CTGF, CCND1, and GPC3 could be used to stratify HCC cases into α2-low and –high groups (bottom panel). Red, over-expression; blue, under-expression; white, no changes.
